# Current clinical care for women with complete androgen insensitivity syndrome across the European reference network on rare endocrine conditions

**DOI:** 10.1007/s40618-026-02990-5

**Published:** 2026-07-20

**Authors:** Eriselda Profka, Alessandra Mangone, Franco Antoniazzi, Federico Baronio, Giuseppe Bellastella, Walter Bonfig, Martine Cools, Régis Coutant, Luisa De Sanctis, Aneta Gawlik-Starzyk, Anna Grandone, Sabine E. Hannema, Olaf Hiort, Paul-Martin Holterhus, Jana Krenek Malikova, Giovanna Motta, Luca Persani, Leonidas Phylactou, Giulia Rastrelli, Stefan Riedl, Vincenzo Rochira, Patrice Rodien, Gianni Russo, Diego Yeste, Martin Wabitsch, Claus H. Gravholt, Hedi Claahsen - van der Grinten, Giovanna Mantovani

**Affiliations:** 1 Department of Clinical Sciences and Community Health, University of Milan, Dipartimento Di Eccellenza, 2023-2027 Milan, Italy; 2 Endocrinology Unit, Fondazione IRCCS Ca’ Granda Ospedale Maggiore Policlinico, 20122 Milan, Italy; 3 Department of Surgery, Dentistry, Pediatrics and Gynecology, Pediatric Clinic C, University of Verona, Verona, Italy; 4 Pediatric Unit, IRCCS Azienda Ospedaliero-Universitaria Di Bologna, Bologna, Italy; 5 Unit of Endocrinology and Metabolic Diseases, Department of Advanced Medical and Surgical Sciences, University of Campania Luigi Vanvitelli, Naples, Italy; 6 Department of Pediatrics, Klinikum Wels, Wels, Austria; 7 Department of Internal Medicine and Paediatrics, Ghent University and Department of Paediatric Endocrinology, Ghent University Hospital, Ghent, Belgium; 8 Pediatric Endocrinology and Diabetology, Reference Center for Rare Diseases of Thyroid and Hormone Receptors, Angers Universital Hospital, Angers, France; 9 Department of Public Health and Pediatric Sciences, University of Torino, Torino, Italy; 10 Department of Pediatrics and Pediatric Endocrinology, Faculty of Medical Sciences, Medical University of Silesia, Katowice, Poland; 11 Department of Woman, Child, General and Specialized Surgery, Università Degli Studi Della Campania Luigi Vanvitelli, Naples, Italy; 12 Dept. of Paediatric Endocrinology, Amsterdam UMC Location Vrije Universiteit, Amsterdam, The Netherlands; 13 Amsterdam Gastroenterology Endocrinology Metabolism, Amsterdam, Netherlands; 14 Amsterdam Reproduction and Development, Amsterdam, Netherlands; 15 University Hospital Schleswig-Holstein, Campus Luebeck, Germany; 16 University Hospital Schleswig-Holstein, Campus, Kiel, Germany; 17 Department of Paediatrics, Second Faculty of Medicine of Charles University and University Hospital Motol, Prague, Czech Republic; 18 Endocrinology, Diabetes and Metabolism, Department of Medical Sciences, University of Turin, Turin, Italy; 19 Dept of Medical Biotechnology and Translational Medicine, University of Milan, Milan, Italy; 20 Department of Endocrine and Metabolic Diseases, RCCS Istituto Auxologico Italiano, Milan, Italy; 21 Department of Molecular Genetics, Function & Therapy, The Cyprus Institute of Neurology & Genetics, Nicosia, Cyprus; 22 Department of Experimental and Clinical Biomedical Science, University of Florence, Careggi Universital Hospital, Florence, Italy; 23 Division of Pediatric Pulmonology, Allergology and Endocrinology, Department of Pediatrics and Adolescent Medicine, Medical University of Vienna, Vienna, Austria; 24 Division of Pediatric Endocrinology, St Anna Children´S Hospital, Medical University of Vienna, Vienna, Austria; 25 Unit of Endocrinology Azienda Ospedaliero, Uiversitaria of Modena, Modena, Italy; 26 Department of Biomedical, Metabolic and Neural Sciences, University of Modena and Reggio Emilia, Modena, Italy; 27 Reference Center for Rare Diseases of Thyroid and Hormone Receptors, Angers University Hospital, Angers, France; 28 Department of Pediatrics, Endocrine Unit, IRCCS San Raffaele Hospital, Milan, Italy; 29 Pediatric Endocrinology Section, Vall d’Hebron University Hospital and Autonomous University of Barcelona, Barcelona, Spain; 30 CIBER Enfermedades Raras, Instituto Carlos III, Madrid, Spain; 31 German Center for Child and Adolescents Health (DZKJ), Partner Site Ulm and Center for Rare Endocrine Diseases, Division of Paediatric Endocrinology and Diabetes, University Medical Center Ulm, Ulm, Germany; 32 Department of Endocrinology, Aarhus University Hospital, Aarhus, Denmark; 33 Department of Clinical Medicine, Aarhus University, Aarhus, Denmark; 34 Department of Pediatrics, Radboud University Medical Center, Nijmegen, Netherlands

**Keywords:** Androgen insensitivity syndrome, Survey, Endo-Ern

## Abstract

**Background:**

Complete androgen insensitivity syndrome (CAIS) is a rare condition affecting sex development. Due to limited literature, especially for providing care in adulthood, clinical management remains challenging, and several issues remain inadequately addressed.

**Methods:**

We conducted an international survey to examine current clinical practices in the management of CAIS across the Referral Centres (RC) of Main Thematic Group 7 (MTG7) of the European Reference Network on Rare Endocrine Conditions (Endo-ERN), with the aim of identifying needs for standardization and potential gaps in care. We collected responses from 24 RC in 11 countries for a total of 256 individuals with CAIS. The majority of respondents were paediatric centres (62.5%), highlighting the challenges in obtaining comprehensive data on adults with CAIS. The survey addressed various aspects of care, including diagnosis, genetic testing, gonadectomy, hormone replacement therapy (HRT), bone health, management of vaginal hypoplasia, and sexual outcomes.

**Results:**

Key findings highlight significant variability in HRT protocols across centres, especially in adulthood, and reveal a lack of standardization in assessing potential long-term outcomes such as bone and sexual health.

**Conclusions:**

Given the complexity and rarity of CAIS, a centralized approach referring patients to centres with expertise in the management of the condition and the development of a clinical practice expert opinion for the management of CAIS beyond the paediatric age could help address current gaps, particularly in the transition from paediatric to adult care. All participating experts emphasized the need to develop such document to optimize CAIS care.

**Supplementary Information:**

The online version contains supplementary material available at https://doi.org/10.1007/s40618-026-02990-5.

## Introduction

Complete androgen insensitivity syndrome (CAIS) is a rare recessive X-linked genetic condition characterized by a female development in 46,XY individuals despite functional testes [1]. It is the most frequent form of 46,XY difference of sex development (DSD), with a prevalence of 1–4.1/20,000–100,000 live births [1–4]. CAIS is caused by loss-of-function variants in the androgen receptor (AR) gene, which is located on the long arm of the X chromosome (Xq11-12) [1–3].

Individuals with CAIS have a typical female appearance of the external genitalia and a short vagina with a blind ending; internal reproductive structures are generally absent due to the regression of the Müllerian structures and lack of androgen-dependent structures, although epididymis or vas deferens have been described [5]. Testicles are present in the labia majora, in the inguinal canal, or more frequently intra-abdominally [1] The onset of puberty is generally spontaneous, with typical female breast development due to the peripheral conversion/aromatization of androgens into oestrogens; however, pubic and axillary hair is sparse or absent. Today, the diagnosis of CAIS is often suspected prenatally due to discordance of a prenatally obtained 46,XY karyotype and the female appearance of the foetus on ultrasound, during childhood due to inguinal hernia with gonadal tissue or during puberty due to primary amenorrhea given the absence of the Müllerian organs. Following clinical and biochemical suspicion, a definitive diagnosis is established through genetic analysis of the AR gene.

The only available guidelines for the care of individuals with CAIS focus on pubertal induction [6], hence in the absence of comprehensive, condition-specific recommendations, clinical management remains challenging even for experienced specialists. Several issues remain controversial, including the management of gonads and the indications for hormonal replacement therapy (HRT). Historically HRT has involved oestrogen therapy with oestradiol, though testosterone is increasingly considered for more physiological replacement [7]. Furthermore, long-term outcomes in adults with CAIS, such as bone and sexual heath, are still largely unexplored [8] although mortality seem to be similar to that of the background population [9].

In the present paper, we report the results of a survey on the current clinical practices in care for women with CAIS across the Reference Centres (RCs) of Main Thematic Group 7 (MTG7) of the European Reference Network on Rare Endocrine Conditions (Endo-ERN). The Endo-ERN was established to address the complexities of rare endocrine diseases by promoting cooperation and sharing knowledge throughout Europe in order to standardize clinical practices, improve patient outcomes, and provide comprehensive care through a network of specialized reference centres (www.endo-ern.eu).

The aim of the present survey was to explore both the variability and commonalities in management approaches across different centres, to identify areas requiring standardization and highlight potential gaps in current clinical practice, with the goal of improving care and outcomes for individuals with CAIS.

## Materials and methods

An international survey was circulated among the RCs of MTG7 within Endo-ERN in spring 2024. The MTG7 is dedicated to the DSD and the Disorders of sex maturation and comprises 53 RCs across Europe, in collaboration with patients’ representatives. Endo-ERN steering committee officially approved this survey as part of its clinical research activities. Responses from Endo-ERN representatives were collected up to May 2024.

Both adult and paediatric endocrinologists listed as Endo-ERN MTG7 representatives for the respective expert centres were eligible to participate. Only one response per RC was allowed. After an initial email, one reminder was sent to non-respondents, approximately 4 weeks following the initial e-mail.

The survey did not contain personally identifiable data of patients (see Supplemental Materials) and questions were aimed to collect data to determine current clinical practices for evaluating individuals affected by CAIS in European RCs.

The survey included 27 questions presented in open-ended and closed format (both binary and multiple choice). The questions were designed to provide information on various clinical aspects of care for women who have CAIS, and included (a) the number of patients followed at the RC and the presence of a multidisciplinary team, (b) diagnostic approach and genetic testing, (c) gonadectomy indications and follow-up of retained gonads, (d) HRT, (e) bone health management, (f) management of vaginal hypoplasia, (g) psychosexual functioning and (h) the need of specific guidelines. Given that the study targeted physicians with specific expertise in the condition, all diagnostic criteria and therapeutic definitions were considered as previously known and were not further detailed in the questionnaire.

All survey responses were analyzed at the center level. For questions with categorical options, the results are presented as the proportion responding centres adopting a given practice or reporting a specific characteristic, calculated using the total number of responding RCs as the denominator. For questions requiring a numerical percentage response, the results were analyzed using descriptive statistics and are presented as mean ± standard deviation. No individual patient data were collected or analysed.

Following the circulation of the manuscript and the feedback received from the participating RCs, some data were revised and supplemented. Final data were accordingly updated in agreement with the contributing participants.

## Results

### Patients and centres

**Fig. 1:**
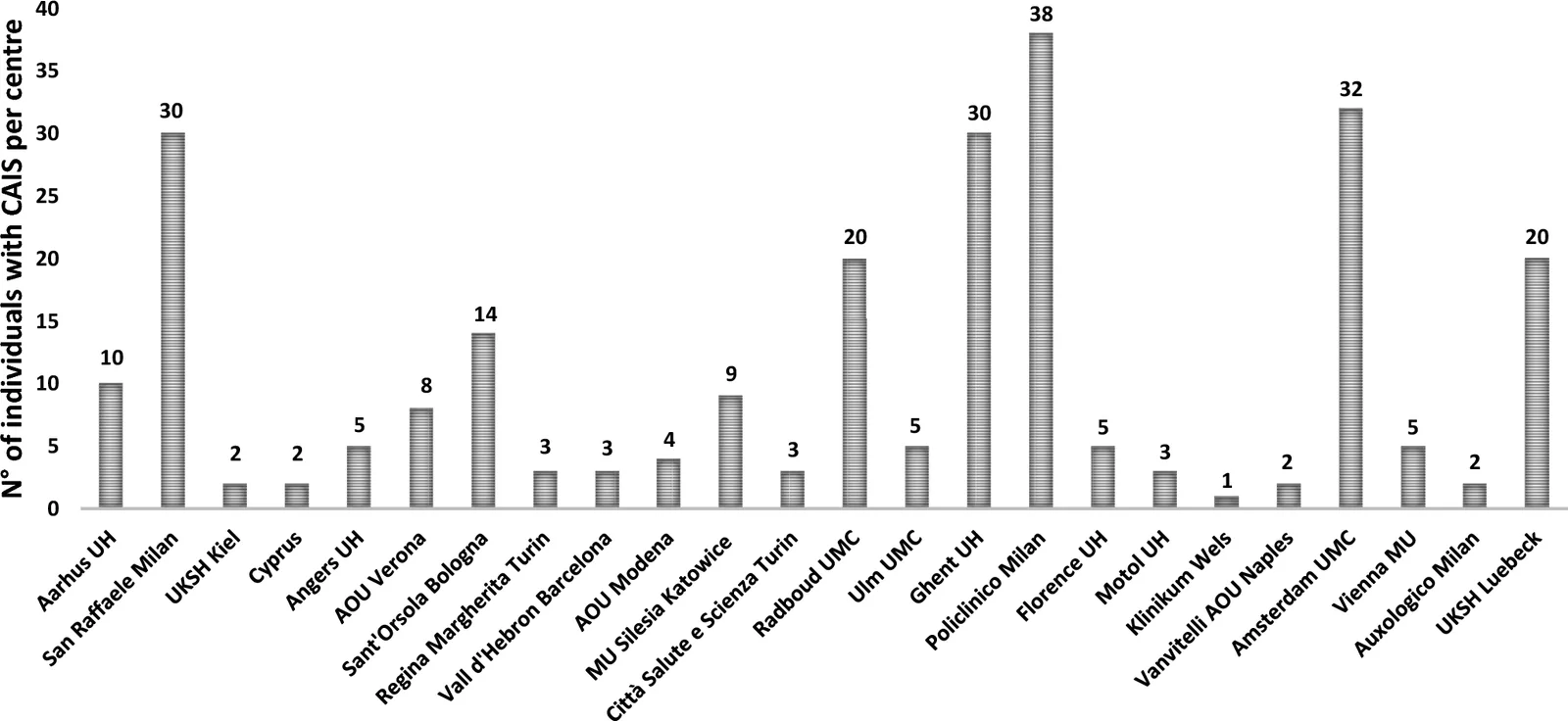
List of participating centres with number of individuals with CAIS followed up

In total, 24 out of 53 MTG7 RCs from 11 countries responded to the survey, as reported in Fig. 1, resulting in a response rate of 45%. Several countries were represented by more than one RC (9 from Italy, 3 from Germany, 2 from the Netherlands, 2 from Austria). The majority (62.5%) of RCs were paediatric centres, 25% were adult centres and 3 centres (12.5%) provided data on both adult and paediatric patients. Overall, we collected data on 256 individuals with CAIS, of whom 169 were currently followed in paediatric care services and 87 were followed in adult care services. The median number of individuals with CAIS followed by each centre was 5 (range 1–38).

All the RCs involved have a dedicated multidisciplinary facility for DSD care in their centre, most of them including paediatric endocrinologists, urologists, gynaecologists, geneticists, and psychologists.

### Genetic testing

There was broad consensus on the need to perform AR gene testing to confirm the diagnosis of CAIS (92% of centres), even when the clinical phenotype, biochemical work-up and karyotype suggest this diagnosis. Moreover, one RC reported performing analysis of the AR target gene apolipoprotein D (APOD) in cases where AR sequencing is normal but the phenotype is strongly suggestive [10, 11]. Segregation analysis in female relatives is performed routinely in 58% of centres, the remaining 42% reported to do this only if desired by the family.

### Management of gonads

With respect to gonadectomy, most RCs (79%) currently perform this procedure only after the patient reaches adulthood However, 8.3% of centres (both adults) still recommend it as soon as possible due to the perceived oncological risk even in paediatric patients. The remaining RCs adopt an individualized approach, discussing the risks and benefits of gonadectomy with adolescents for shared decision-making; in these cases, the RCs underline how most or all of them choose to preserve the gonads. A mean of 28.7 ± 32% individuals had undergone gonadectomy before completing pubertal development, whereas 34.9 ± 38% had the procedure afterwards. At the time of the survey, a mean of 34.5 ± 38% individuals still have gonads in situ, 16% considering centres following adults.

**Fig. 2:**
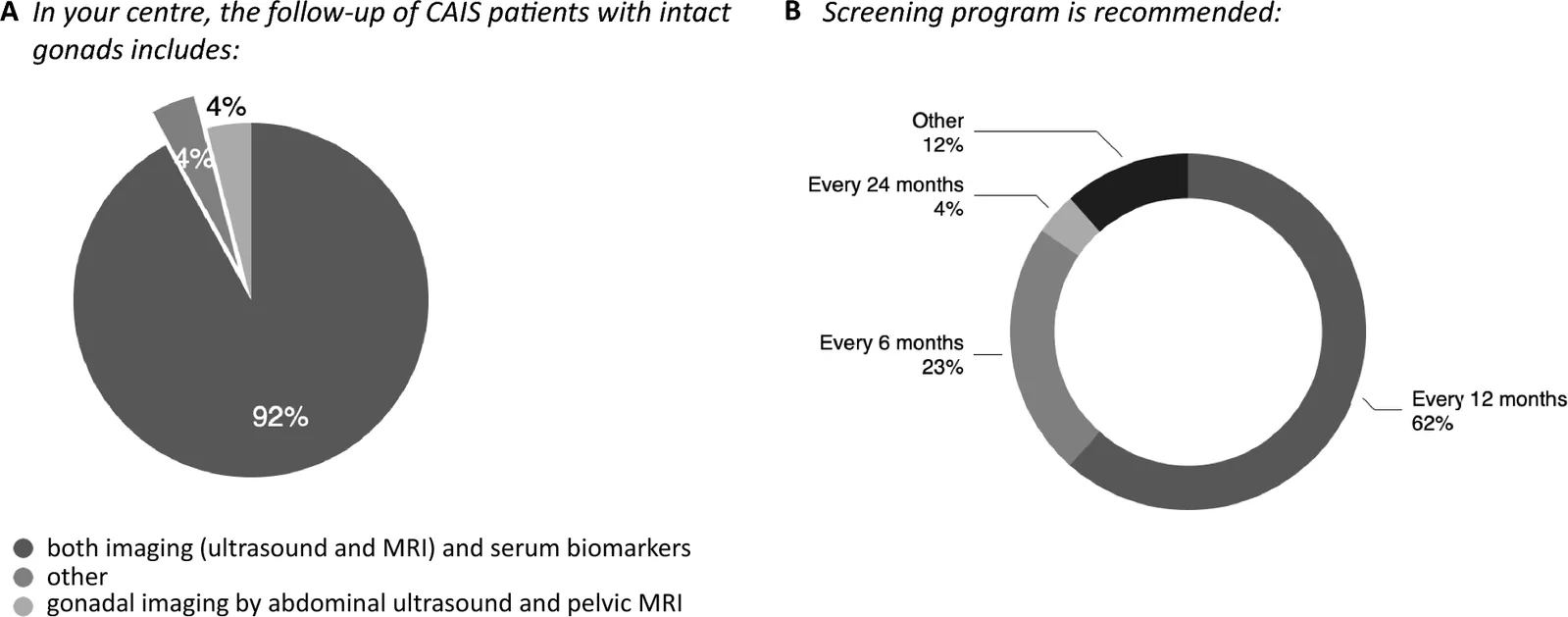
Screening programs of intact gonads according to the participating centres. Legend: MRI = Magnetic Resonance Imaging. Serum biomarkers includes α-fetoprotein, β-human chorionic gonadotropin, lactate dehydrogenase, placental-like alkaline phosphatase in non-smokers and hormone blood tests (LH, FSH, testosterone, oestradiol); 2A: Other answers include: Not applicable or serum biomarkers + annual MRI only. 2B: Other answer include: Not applicable, screening only after 16 years old or annual imaging and semesterly serum biomarkers

In cases of retained gonads, a screening program is suggested, which includes a combination of imaging techniques (abdominal ultrasound and pelvic magnetic resonance—MRI) and serum biomarkers for testicular tumours (α-fetoprotein, β-human chorionic gonadotropin, lactate dehydrogenase, placental-like alkaline phosphatase in non-smokers) and hormone blood tests in 92% of RCs (see Fig. 2A). As for the timing, 60% suggest a screening program every 12 months, 24% every 6 months (Fig. 2B).

### Hormonal therapy

In case gonadectomy was performed before puberty, most RCs start HRT between the ages of 10.5 and 12 years (54% 10.5–11 years, 33% 11–12 years, 13% after 12 years). Puberty is induced with transdermal oestradiol in most centres (62.5%), oral oestradiol valerate (21%) or oral 17β oestradiol (12.5%), rarely with ethinyl oestradiol (4%).

HRT regimens prescribed for maintenance in RCs, along with the relative percentage of patients receiving each formulation per centre, are summarized in Table 1. Transdermal oestrogen formulation seems to be preferred in most centres, although oral oestradiol is also widely prescribed. There are also centres using a formulation of combined oestrogens with progestins. Three RCs are prescribing testosterone to adults with CAIS: 2 using the transdermal formulation, and one either transdermal or intramuscular formulations.

**Table 1:** Percentage of patients in each Centre on different HRT regimens after gonadectomy

Centre	Adult or paediatric centre	N° of CAIS	Hormonal replacement therapy (HRT) regimes					
			Transd. E2	Oral E2	Oral EE	Oral EP	Testost	Testost. + E2
1	A	10	10%	90%	0%	0%	0%	0%
2	P	30*	40%	40%	0%	0%	0%	0%
3	P	2	*NA*	*NA*	*NA*	*NA*	*NA*	*NA*
4	P	2	50%	0%	50%	0%	0%	0%
5	P	5	*NA*	*NA*	*NA*	*NA*	*NA*	*NA*
6	P	8	90%	0%	0%	0%	0%	0%
7	P	14	0	60%	0%	40%	0%	0%
8	P	3	*NA*	*NA*	*NA*	*NA*	*NA*	*NA*
9	P	3	*NA*	*NA*	*NA*	*NA*	0%	0%
10	A	4	100%	0%	0%	0%	0%	0%
11	P	9	75%	25%	0%	0%	0%	0%
12	A	3	67%	0%	0%	33%	0%	0%
13	A + P	20*	10%	10%	0%	0%	0%	0%
14	P	5*	0%	0%	0%	0%	0%	0%
15	P	30	37.5%	62.5%	0%	0%	0%	0%
16	A	38	54.9%	32%	0%	0%	10.5%	2.6%
17	A	5	60%	0%	0%	0%	20%	0
18	P	3	*NA*	*NA*	*NA*	*NA*	*NA*	*NA*
19	P	1	*100%*	0%	0%	0%	0%	0%
20	A + P	2	100%	0%	0%	0%	0%	0%
21	A + P	32	*NA*	*NA*	*NA*	*NA*	*NA*	*NA*
22	P	5*	20%	0%	0%	0%	0%	0%
23	A	2	100%	0%	0%	0%	0%	0%
24	P	20	0%	5%	0%	0%	10%	0%

A: adult; P: paediatric; N: number; E2: Oestradiol; EE: ethinyl estradiol; EP: oestradiol + progesterone; Transd. E2: transdermic formulation of oestradiol; Oral E2: oral oestradiol valerate; Oral EE: oral formulation with ethinyl estradiol; Oral EP: combined oestrogen-progestogen oral contraceptive pill; Testost.: testosterone; Testost. + E2: testosterone and oestrogen association; *NA*: not available data. *the remaining percentage of patients are not receiving any therapy at the moment

Finally, we asked RCs until what age HRT is usually prescribed: 54.2% reported prescribing it until the natural average age of menopause, whereas the remaining RCs consider extending it further if requested by the concerned individuals.

### Long-term outcomes

Focusing on bone health assessment, 66.6% of RCs report routinely performing a Dual-energy X-ray Absorptiometry (DEXA), while 8.3% only perform it in gonadectomized individuals. However, bone mass data were limited, as only 12 centres (50%), including 6 managing adults, provided this information, restricting interpretation. From the available data, we can derive on average a prevalence of osteopenia of 26.5 ± 24% and of osteoporosis of 8.2 ± 15%. When considering only centres following adults, these percentage increase to 37.3 ± 29% and 13 ± 20% of osteopenia and osteoporosis, respectively.

When bone mass impairment is diagnosed, most (83%) RCs prescribe vitamin D supplementation, and 75% also recommend supplemental calcium intake. On average, 45.1 ± 41% of women are receiving supplemental vitamin D and 27.7 ± 36% also calcium, whereas only 3 adult RCs (12.5%) prescribed bisphosphonates, and 1 other anti-absorptive therapies.

When analysing the approach to vaginal hypoplasia, 66.6% of RCs recommend non-surgical vaginal self-dilatation as the first-line treatment. Across the centres, the mean reported percentage of individuals undergoing the procedure is 26% overall and 31.2 ± 37% considering adult centres only. On the contrary, 4.2% of centres initially propose laparoscopic vaginoplasty, whereas in 29% of RCs the laparoscopic procedure is proposed to women who have difficulty adhering to, do not achieve adequate outcomes with, or prefer not to initiate vaginal dilation.

Finally, we asked whether RCs assess sexual health in individuals with CAIS using specific questionnaires. Seven (29%) centres report doing so, 4 of which being adult centres (see Table 2), and the tools used are heterogeneous, with the Female Sexual Function Index (FSFI) being the most applied (4 centres).

**Table 2:** Responses of the centres who reported to investigate sexual function and tools employed

Adult or paediatric centre	Do you assess sexual function with specific questionnaires?	If yes, what kind of questionnaire do you use?
A	Yes	*Not specified*
P	Yes	Personal communication
A	Yes	FSFI
P	No	FSFI in case of research
A	Yes	FSFI; BUT and FSDS-R
P	Yes	We have a specifically trained nurse in sexual heath
A + P	Yes	FSFI

A: adult; P: paediatric; FSFI: Female Sexual Function Index; BUT: Body Uneasiness Test; FSDS-R: 13-item Female Sexual Distress Scale-Revised

All but one RCs agreed on the need to elaborate standardized guidelines/expert opinion for managing women who have CAIS.

## Discussion

The present survey investigates current clinical practices for the management of individuals with CAIS across Endo-ERN RCs. The rarity of the condition poses several challenges, and indeed the first aspect emerging from our work relies on the limited number of individuals actively followed, even in the concerned RCs. Although the survey targeted expert centres, only 25% reported following more than 15 individuals with CAIS. Moreover, the majority of responding RCs were paediatric centres, resulting in limited data on adults and, consequently, on long-term management and outcomes, areas where current knowledge remains particularly scarce. Moreover, current medical approaches are often shaped by the categorization of individuals with CAIS within the female framework. While this aligns with the phenotypic presentation and gender identity of most individuals, it may not fully account for certain clinical specificities, particularly in areas such as hormonal replacement and bone health, where condition-specific reference values are lacking and evidence-based recommendations are still insufficient. The limited number of adult RCs also may raise concerns regarding the possibilities for transition of adolescents from paediatric to adult care, potentially indicating a gap in current clinical practice. It may be important, within the Endo-ERN community, to promote projects aimed at improving the transition process and at centralizing care in experienced centres following larger cohorts to ensure continuous and comprehensive high-quality management of CAIS throughout the patient's life.

Consistent with the holistic approach advocated by DSD experts [12], all RCs confirmed having a dedicated multidisciplinary facility for DSD care. Most RCs acknowledge the importance of genetic testing for the diagnosis of CAIS, which contributes to a more accurate characterization of the condition.

The indication and timing of gonadectomy in individuals with CAIS remain controversial in the scientific community. Most RCs recommend gonadectomy only after the individual has reached adulthood, in line with the principle of shared informed decision-making and respect for patient autonomy, yet a minority of RCs still advocates for early gonadectomy; however, given the limited sample size, this finding should be interpreted with caution and may reflect centre-specific clinical perspectives. The remaining healthcare professionals adopt a personalized approach, engaging in discussions with adolescents or adults [13]. Although undescended testes have historically been correlated with an increased risk of testicular germ cell tumours, recent evidence suggests that the oncological risk in prepubertal girls with CAIS is very low [13–16], supporting conservative management at least until full pubertal development [13]. In adults invasive germ cell cancers are also very rarely reported [13, 16] and a relatively large study in post pubertal individuals with CAIS did not identify a single case of invasive germ cell cancer [17]. This low risk in AIS has been attributed to the lack of androgenic action, since androgen exposure is considered a major risk factor for the progression of in situ pre-neoplastic lesions towards invasive cancer [18]. The advantage of a gonad-sparing approach is the potential to avoid HRT, thereby reducing the medicalization of the condition. If this conservative approach results in better preservation of bone mass remains to be determined. When a gonad-sparing strategy is chosen, and in the absence of more long-term outcome data on invasive germ cell cancer risk, regular follow-up is essential to ensure early detection of malignant transformation. A standardized monitoring protocol combining imaging and serum biomarkers [12, 17, 19, 20] has been proposed [13], and our results demonstrate that nearly all RCs have adopted this protocol. We registered greater variability in screening frequency, with annual evaluations resulting generally preferred. Unfortunately, data on the detection of malignant tumours in the RCs were not collected in this survey, and are therefore unavailable.

HRT is essential following gonadectomy and most RCs prescribe oestradiol therapy according to existing protocols for female hypogonadism and the proposed guidelines for CAIS [6], preferably using the transdermal formulation but individualized based on patient preference. Progestin treatment is not recommended by guidelines given the absence of a uterus and possible negative effects on bone health [15, 21–23]. In recent years, testosterone has emerged as an alternative HRT for CAIS, supported by a multicentre double-blind cross-over study showing potential benefits in terms of sexual desire compared to oestrogens [7], with no significant differences in metabolic profile [24]. Our results on the limited prescription of testosterone in clinical practice (3 adult RCs only) suggest that its clinical use is not yet widespread. The issue of compliance with HRT should also be considered. As for the duration of therapy, half of the RCs prescribe HRT until the average age of menopause, while the others consider extending it beyond this point. This variability highlights another area in need of further investigation.

Finally, we were interested in exploring the management of long-term outcomes in CAIS among experts, an area especially lacking robust clinical evidence. However, as stated above, this objective was limited by the small adult sample size. Despite all RCs agreed on the importance of bone health screening, only a small subset could provide comprehensive data on bone density measurements in their cohorts. Consequently, the real prevalence of bone mass impairment in CAIS remains incompletely defined and will need to be better explored, particularly given its high incidence in literature despite the young age cohorts [21, 25, 26], possibly linked to androgen resistance itself as well as the hormonal deficiency which follows gonadectomy [27–32]. In a nationwide study, fractures also resulted more frequent among women with CAIS [9]. Moreover, the substantial lack of data on therapeutic strategies in this population complicates clinical management and underscores the need for further research.

Regarding the management of vaginal hypoplasia, non-surgical vaginal dilation has been recommended as the first-line therapy in individuals with CAIS [33]. However, as reflected in our findings, the implementation of this recommendation remains inconsistent across centres.

A lack of sexual confidence and reduced sexual satisfaction have been reported in women with CAIS [34–39], but only a few RCs report to systemically explore this aspect. We believe that sexual health deserves greater attention in future studies, as it plays a fundamental role in long-term psychological well-being and need to be addressed to enable appropriate interventions, such as the inclusion from adolescence of a sexologist in the multidisciplinary team.

In summary, the management of CAIS remains complex due to the rarity of the condition and the limited data, particularly in the adult population. While multidisciplinary care and genetic testing are well established, several aspects including optimal timing of gonadectomy, HRT protocols, and long-term outcomes such as bone health and sexual well-being require further investigation. At the same time, we observe that available guidelines on these matters are not uniformly implemented, a problem that urgently needs to be addressed.

This study has inherent limitations due to its survey-based design and should be considered as a preliminary effort aimed at providing a snapshot of current clinical practice and generating hypotheses for future research. The relatively modest response rate may have introduced response bias. In addition, the predominance of paediatric centres limits the representation of adult care, while the centre-level analysis precludes patient-level insights. Larger collaborative studies, including a broader representation of adult patients, are needed to better characterize clinical practice across the lifespan and to inform the development of future recommendations.

In conclusion, this complex and rare condition would greatly benefit from a more centralized approach, and by addressing the current gaps in the transition from paediatric to adult care. The choice and management of HRT should be individualized, considering individuals’ preferences alongside careful evaluation of long-term outcomes, such as bone health and sexual function. The unanimous consensus on the need to develop expert opinion for the management of individuals with CAIS further underscores the urgency of collaborative efforts within the Endo-ERN network (e.g. through SDM registries) to improve care and to ensure evidence-based, patient-centred management. 

## Supplementary Information

Below is the link to the electronic supplementary material.

Supplementary file1

## Data Availability

The datasets generated and/or analysed during the current study are available from the corresponding author on reasonable request, subject to agreement from the participating centres.
