# Supplementary material for: Current clinical care for women with complete androgen insensitivity syndrome across the European reference network on rare endocrine conditions
Source: J Endocrinol Invest. 2026 Jul 20;49(11):3399–407. doi: 10.1007/s40618-026-02990-5 (PMC13722754; doi:10.1007/s40618-026-02990-5)
Supplement: Supplementary file 1 [file 40618_2026_2990_MOESM1_ESM.pdf]

SURVEY on diagnosis and management of complete androgen insensibility syndrome (CAIS)

Complete androgen insensibility syndrome (CAIS) is the most common form of 46 XY differences of sex development (DSD). Although CAIS etiopathogenesis have been well-defined, several issues remain controversial and the long-term outcomes are largely unexplored.

1) **In which country do you practice?________________**

2) **Please indicate the name of your center/ your affiliation:**
   ____

3) **Please indicate your profession/position (please indicate the term which best describes you):**
- pediatric endocrinologist
- adult endocrinologist
- pediatrician with an interest in endocrinology
- other, specify___________

4) **How many CAIS do you follow in your center?**
   -please specify the total number

5) **In your center, do you have a multidisciplinary team (including pediatric endocrinologists, urologists, gynecologists, geneticists, and psychologists) for the management of CAIS patients?**
   - Yes
   - No
   - Other, specify_____

6) **In your center, does the definitive diagnosis of CAIS require the execution of the genetic test on AR gene?**
   -Yes, it is necessary for the diagnosis

-No, it is not necessary if karyotype is 46XY in female patients

-other, specify____

7) **Considering the X-linked transmission of CAIS and its possible recurrence within the same family, do you perform a genetic screening on the proband's mother and, whether positive, on her female relatives?**

-Yes, in all patients

-No

-Only if requested by the family

8) **In your center, presently gonadectomy is performed:**

- as soon as the diagnosis is achieved (for possible oncological risk)
- prior to complete pubertal development
- after complete pubertal development
- only after the achievement of the age of maturity, permitting a spontaneous growth spurt and an autonomous decision about surgery

-other, specify____

9) **In your center, how many of CAIS patients underwent gonadectomy (%):**

- as soon as the diagnosis was achieved:__________
- prior to complete pubertal development:__________
- after completed pubertal development:__________
- gonadectomy not performed: ________________

10) **In your center, the follow-up of CAIS patients with intact gonads includes:**

- gonadal imaging by abdominal ultrasound and pelvic magnetic resonance
- serum biomarkers of testicular tumors (α-fetoprotein, β-hCG, LDH), and hormone blood tests (LH, FSH, testosterone)
- both imaging by abdominal ultrasound and pelvic magnetic resonance and serum biomarkers of testicular tumors and hormone blood tests

-other, specify____

11) **In your center, in CAIS patients with intact gonads, screening program is recommended:**

- every 6 months

- every 12 months
- every 24 months
- other, specify____

12) **In your center, in patients who have undergone gonadectomy before puberty, at what age do you start hormonal replacement therapy?**

- 8-10 years
- 10.5-11 years
- ≥ 12 years
- other, specify _______

13) **Adequate hormonal replacement therapy should be gradually administered to adolescents who had undergone gonadectomy before puberty to provide a harmonic pubertal growth spurt, optimal bone mineral accumulation, appropriate psychosexual maturation, and psychosocial well-being:**

-I agree

-I disagree

-other, specify ________________

14) **What kind of estrogens do you use as first-line therapy to induce puberty?**

- transdermal estradiol
- oral estradiol valerate
- oral ethinyl estradiol

-other, specify________________

15) **In your center how many of adult CAIS patients (%) are treated with:**

- transdermal estradiol: ____________
- oral estradiol valerate:_____________
- oral ethinyl estradiol:_________________
- combined estrogens+progestins:____________
- testosterone__________________
- testosterone in combination with estrogens:_____________

-no hormone treatment:___________

-other, specify________________

16) What preparation of testosterone do you generally use? (you can choose more than one)

-transdermal testosterone

- intramuscolar testosterone

-other, please specify

-we do not use testosterone in CAIS patients

17) In your center, hormonal replacement therapy is prescribed until the average age of natural menopause:

-yes

-no

-other, specify_______________

18) In your center, do you perform lumbar and femoral Dual-energy X-ray absorptiometry (DEXA) in CAIS patients?

-yes, in all CAIS patients

-only in CAIS who underwent gonadectomy

-only in CAIS with fragility fractures

-other, specify_______________

19) In your center, how many of CAIS patients (%) have:

- osteopenia:_______________

- osteoporosis:_____________

- normal bone mineralization: ____________

- not evaluated:________________

20) In your center, in CAIS with osteopenia or osteoporosis do you prescribe supplemental calcium and vitamin D intake?

-yes, in all patients

-no

-other, specify ____________

21) In your center, how many of CAIS patients (%) are treated with:

- supplemental calcium intake _______________
- supplemental vitamin D intake _______________
- biphosphonates:___________
- other:______
- none of the above:_______________

22) CAIS patients usually have a short vaginal dimple of 2.5-8 cm in length, ending in a blind bottom. What is the therapeutic attitude in these patients in your center?

- we suggest non-surgical vaginal self-dilations as first treatment option in CAIS patients with hypoplastic vaginas
- we suggest the laparoscopic Vecchietti's procedure as the most appropriate procedure
- we suggest the laparoscopic Vecchietti's procedure as the most appropriate surgical strategy in specialized centers to treat patients who are poorly compliant or failed or do not want to start a vaginal dilation therapy.

23) How many of your CAIS patients have needed non-surgical vaginal self-dilations?

___________________(%)?___

24) How many of your CAIS patients have needed laparoscopic Vecchietti's procedure?

__________________________(%)?___

25) Do you assess sexual function with specific questionaries in your CAIS patients?

- Yes
- No

26) If yes, what kind of questionnaire do you use?

____

27) Do you think that standardized international guidelines should be elaborated for CAIS patients?

- Yes
- No
